# Application of artificial intelligence in Alzheimer’s disease: a bibliometric analysis

**DOI:** 10.3389/fnins.2025.1511350

**Published:** 2025-02-14

**Authors:** Sijia Song, Tong Li, Wei Lin, Ran Liu, Yujie Zhang

**Affiliations:** ^1^School of Intelligent Medicine, Chengdu University of Traditional Chinese Medicine, Chengdu, China; ^2^School of Biomedical Engineering, Tsinghua University, Beijing, China

**Keywords:** artificial intelligence, Alzheimer’s disease, machine learning, bibliometric analysis, VOSviewer, CiteSpace

## Abstract

**Background:**

Understanding how artificial intelligence (AI) is employed to predict, diagnose, and perform relevant analyses in Alzheimer’s disease research is a rapidly evolving field. This study integrated and analyzed the relevant literature from the Science Citation Index (SCI) and Social Science Citation Index (SSCI) on the application of AI in Alzheimer’s disease (AD), covering publications from 2004 to 2023.

**Objective:**

This study aims to identify the key research hotspots and trends of the application of AI in AD over the past 20 years through a bibliometric analysis.

**Methods:**

Using the Web of Science Core Collection database, we conducted a comprehensive visual analysis of literature on AI and AD published between January 1, 2004, and December 31, 2023. The study utilized Excel, Scimago Graphica, VOSviewer, and CiteSpace software to visualize trends in annual publications and the distribution of research by countries, institutions, journals, references, authors, and keywords related to this topic.

**Results:**

A total of 2,316 papers were obtained through the research process, with a significant increase in publications observed since 2018, signaling notable growth in this field. The United States, China, and the United Kingdom made notable contributions to this research area. The University of London led in institutional productivity with 80 publications, followed by the University of California System with 74 publications. Regarding total publications, the *Journal of Alzheimer’s Disease* was the most prolific while *Neuroimage* ranked as the most cited journal. Shen Dinggang was the top author in both total publications and average citations. Analysis of reference and keyword highlighted research hotspots, including the identification of various stages of AD, early diagnostic screening, risk prediction, and prediction of disease progression. The “task analysis” keyword emerged as a research frontier from 2021 to 2023.

**Conclusion:**

Research on AI applications in AD holds significant potential for practical advancements, attracting increasing attention from scholars. Deep learning (DL) techniques have emerged as a key research focus for AD diagnosis. Future research will explore AI methods, particularly task analysis, emphasizing integrating multimodal data and utilizing deep neural networks. These approaches aim to identify emerging risk factors, such as environmental influences on AD onset, predict disease progression with high accuracy, and support the development of prevention strategies. Ultimately, AI-driven innovations will transform AD management from a progressive, incurable state to a more manageable and potentially reversible condition, thereby improving healthcare, rehabilitation, and long-term care solutions.

## 1 Introduction

AD is a progressive and irreversible neurological disorder and the leading cause of dementia (Gaugler et al., 2019). It’s pathological features significantly impair patients’ cognitive functions and daily activities, severely diminishing their quality of life. As the global elderly population continues to rise, the number of AD cases is increasing correspondingly (Deng et al., 2023). By 2025, the prevalence of AD is expected to double, making it one of the most burdensome diseases of this century (Scheltens et al., 2021). According to data and statistics on AD in 2020, it is projected that the population of individuals aged 65 and older in the United States with AD will increase significantly, from 5.8 million currently to 13.8 million by 2050. A similar trend has been observed in Japan, where community surveys conducted over the past few decades have shown a notable rise in the prevalence of AD (Ohara et al., 2017). Likewise, in China, community surveys over the past few decades indicate a substantial increase in the prevalence of AD (Chan et al., 2013). Additionally, carers face increased psychological stress and negative emotions, placing a heavy and unsustainable burden on both society and families. The disease not only causes immense physical and psychological distress for patients but also places a substantial emotional and financial strain on families and society (Gwyther, 1998). At the same time, the diagnosis and treatment of AD face significant challenges. AD’s insidious onset makes early diagnosis particularly difficult. By the time clinical symptoms become apparent, the disease has often reached an irreversible stage, complicating early intervention and treatment. Despite recent advances in biomarker testing and neuroimaging, the diagnosis of AD still largely depends on clinical presentation and neuropsychological assessment. As a result, there is a clear need to improve the accuracy and reliability of these methods. Additionally, the pathogenesis of AD is highly complex, involving interactions among multiple neurotransmitters, apoptosis, inflammatory responses, and various genetic and environmental factors (Khan et al., 2020). Despite extensive research efforts, there are still no effective therapeutic strategies for alter or halt the progression of AD (Fabrizio et al., 2021). Current treatments can only alleviate symptoms. Consequently, comprehensive research on the prevention, diagnosis, and treatment of AD, along with developing new therapeutic approaches and technologies, remains an urgent priority in the medical and scientific communities.

In recent years, the remarkable advances in AI technology, along with its interdisciplinary and integrated applications in medicine, have presented promising solutions to a series of critical challenges that have long plagued medical research. Machine learning (ML) and deep learning (DL) are two fundamental subsets of AI (Lu et al., 2018). The core principle of ML is to derive rules from data using algorithms to predict or classify new data. The application of ML to electronic health records is becoming increasingly prevalent in medical research. For example, one research team successfully modeled patients’ disease trajectories and constructed a personalized disease progression prediction model using the Conditional Restricted Boltzmann Machine (CRBM) model and 18 months clinical data (covering changes in 44 clinical variables) from 1,909 patients with mild cognitive impairment (MCI) or AD (Fisher et al., 2019). Moradi et al. (2015) used MRI data along with supervised learning and random forest techniques to develop a novel biomarker for AD, significantly improving diagnostic accuracy and laying the foundation for early intervention. However, one of the challenges of ML, particularly when dealing with large datasets, is its reliance on manual feature extraction—a time-consuming process that adds complexity to data analysis. DL, a branch of ML, overcomes this limitation by employing deep neural networks to simulate the way the human brain processes information. Through multi-layer nonlinear transformations, DL automatically extracts high-level features, making it especially suited for processing large-scale, high-dimensional datasets. The emergence of DL technology has enabled researchers to develop DL-based AD prediction and diagnosis models (Koga et al., 2022; Qiu et al., 2022). These models have shown superior specificity and sensitivity compared to traditional ML methods, offering powerful technical support for early detection and accurate diagnosis of AD. Lahmiri (2023) proposed an automated system that integrates convolutional neural networks (CNNs), which significantly improved the accuracy, sensitivity and specificity of MRI-based diagnosis of AD. Alhudhaif and Polat (2023) proposed a deep learning model supported by a fusion loss function for efficiently classifying the stages of AD based on MRI images. This model achieves high precision in classification and effectively monitors disease progression, assisting physicians in decision-making. Additionally, by utilizing a web-based AI framework that integrates multi-omics data and GWAS results, the researchers successfully identified 103 genes associated with AD risk. Furthermore, they found that three medications, including pioglitazone, were significantly linked to a substantial reduction in AD risk (Fang et al., 2022). In conclusion, AI holds tremendous potential in the field of Alzheimer’s disease, with a wide range of applications. As technology evolves and research progresses, it is expected that AI will continue to make significant breakthroughs, contributing to the advancements in human health.

A bibliometric study has already been conducted on the application of AI in brain diseases (Wu et al., 2023). Building on these findings and given the current research trends, we aim to conduct a more detailed and cutting-edge bibliometric analysis of AI applications in AD, with the goal of uncovering new insights and breakthroughs.

Therefore, this study employs bibliometric methodologies, along with literature visualization tools, to provide a comprehensive analysis of the research on the application of AI in AD globally from 2004 to 2023. The objective is to identify the most productive countries, institutions, journals, and authors, as well as key keywords and co-cited literature. The findings are visually mapped to reveal the research hotspots and future trends in this rapidly evolving field.

## 2 Materials and methods

### 2.1 Search strategy

The Web of Science database is a leading platform for indexing abstracts of interdisciplinary scholarly literature, encompassing over 12,400 high-impact journals. Widely adopted in bibliometric research, it provides essential data for academic evaluation. The data used in this study were sourced from the Web of Science Core Collection, including citation indexes such as the Science Citation Index (SCI) and the Social Science Citation Index (SSCI). The literature search was conducted on May 4, 2024. The term “TS” refers to “Topic Search,” which allows searching within the titles, abstracts, and keywords of publications. The search strategy, based on previous studies, was as follows: TS = (“artificial intelligence” OR “deep learning” OR “machine learning” OR “neural network” OR “natural language processing”) AND TS = (“Alzheimer*”). The search was limited to Articles and Review Articles in English, covering publications from January 1, 2004, to December 31, 2023. To ensure accuracy and reliability, the search process was conducted independently by two researchers.

### 2.2 Inclusion and exclusion criteria

The inclusion criteria were as follows: (1) The research focused on Alzheimer’s disease (AD). (2) The research employed AI-related technologies (such as deep learning, machine learning, neural networks, natural language processing, etc.).

The exclusion criteria were as follows: (1) The research did not focus on AD. (2) The research did not involve AI techniques.

### 2.3 Data collection

After eliminating duplicates and unpublished literature, 2,316 articles were selected for further analysis. For each publication, the following basic data were collected: title, abstract, country or region, institution, author, keywords, journal, and references.

### 2.4 Data analysis and visualization

In this study, we employed CiteSpace (v.6.3.R1 64-bit advanced), VOSviewer (v.1.6.20), and Scimago Graphica (1.0.45) for conducting bibliometric and visual analyses.

CiteSpace, developed by Professor Chen Chaomei, is a specialized visual analysis software designed to identify research hotspots and predict future research trends within the literature (Chen, 2006). Specifically, we utilized CiteSpace for the following tasks: co-occurrence analysis of institutions, dual graph overlay of journals, citation burst analysis of references, and keyword clustering and burst detection. The parameters used in CiteSpace were as follows: Timespan: 2004–2023; Slice Length = 1; Threshold value: g-index (*k* = 25), LRF = 3.0, L/N = 10, LBT = 5, *e* = 1.0.

VOSviewer, developed by the University of Leiden, is designed for constructing and visualizing bibliometric networks. It is particularly effective for analyzing correlations using clustering and co-occurrence methods, revealing research hotspots and collaborative network distributions (van Eck and Waltman, 2010). Using VOSviewer, we performed co-authorship analysis, keyword co-occurrence analysis, and reference co-citation analysis. Additionally, we visualized global co-author networks using both VOSviewer and Scimago Graphica. The parameters set for VOSviewer were as follows: Author: minimum number of documents per author: 6, minimum number of citations per author: 0; Keywords: minimum number of occurrences per keyword: 5; Cited reference: minimum number of citations per reference: 35.

## 3 Results

### 3.1 Research selection process

**Figure 1:**
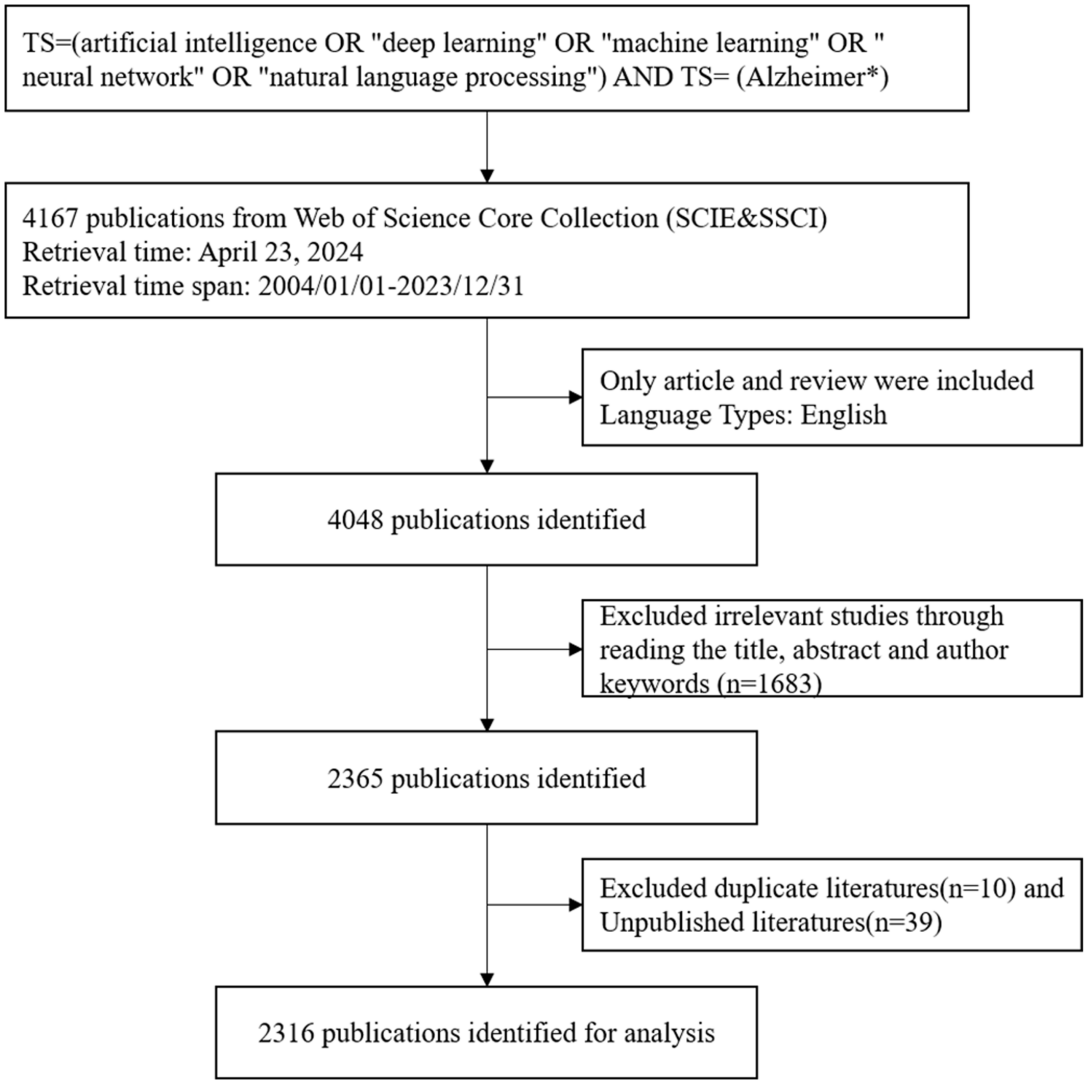
Flow chart of the literature screen.

After conducting the initial search, we screened the titles and abstracts to confirm the eligibility of articles based on predefined inclusion and exclusion criteria. The manual exclusion criteria were: (1) The research did not focus on AD. (2) The research did not involve AI-related methods. The search process was carried out independently by two researchers to maximize the accuracy and reliability of the results. Based on these criteria, 1,683 references were excluded after reviewing the titles and abstracts. Duplicates and unpublished literature were then removed. Finally, 2,316 articles were selected for further consideration. The literature retrieval and screening process is shown in Figure 1.

### 3.2 Global trends of publication outputs and citations

**Figure 2:**
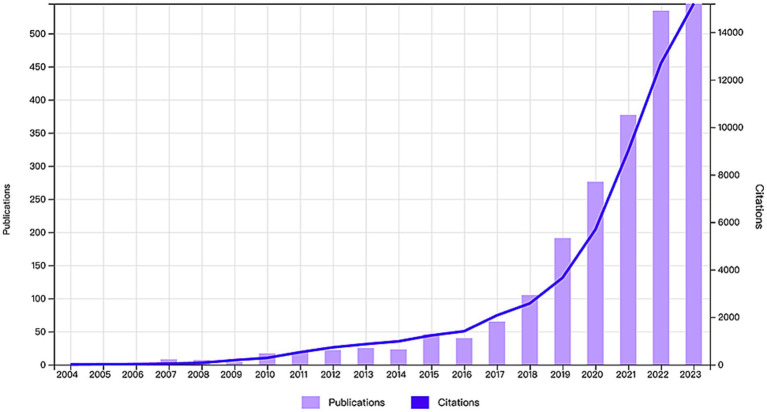
Annual publication volume and annual citation frequency of articles related to the field of AI in AD in the last two decades.

By statistically analyzing the number of published research papers over different periods, we can uncover the research trends and developmental dynamics within a particular field. Based on the established search strategy and screening process, we successfully collected a total of 2,316 research papers related to the application of AI in AD from the WoSCC database over the past 20 years (Figure 1). We then plotted the publication trends over time (Figure 2), which clearly illustrates the significant growth in research on AI applications in Alzheimer’s disease. Specifically, before 2009, research in this area was still in its early stages, with only a few papers being published. However, between 2010 and 2018, the number of publications began to rise gradually. Notably, after 2018, the field experienced exponential growth, reaching a peak of 555 publications in 2023. Additionally, as of the search date, these papers have been cited a total of 57,230 times, with an average of 24.71 citations per paper, underscoring the substantial impact and academic significance of research in this field.

### 3.3 Contributions of countries/regions

**Figure 3:**
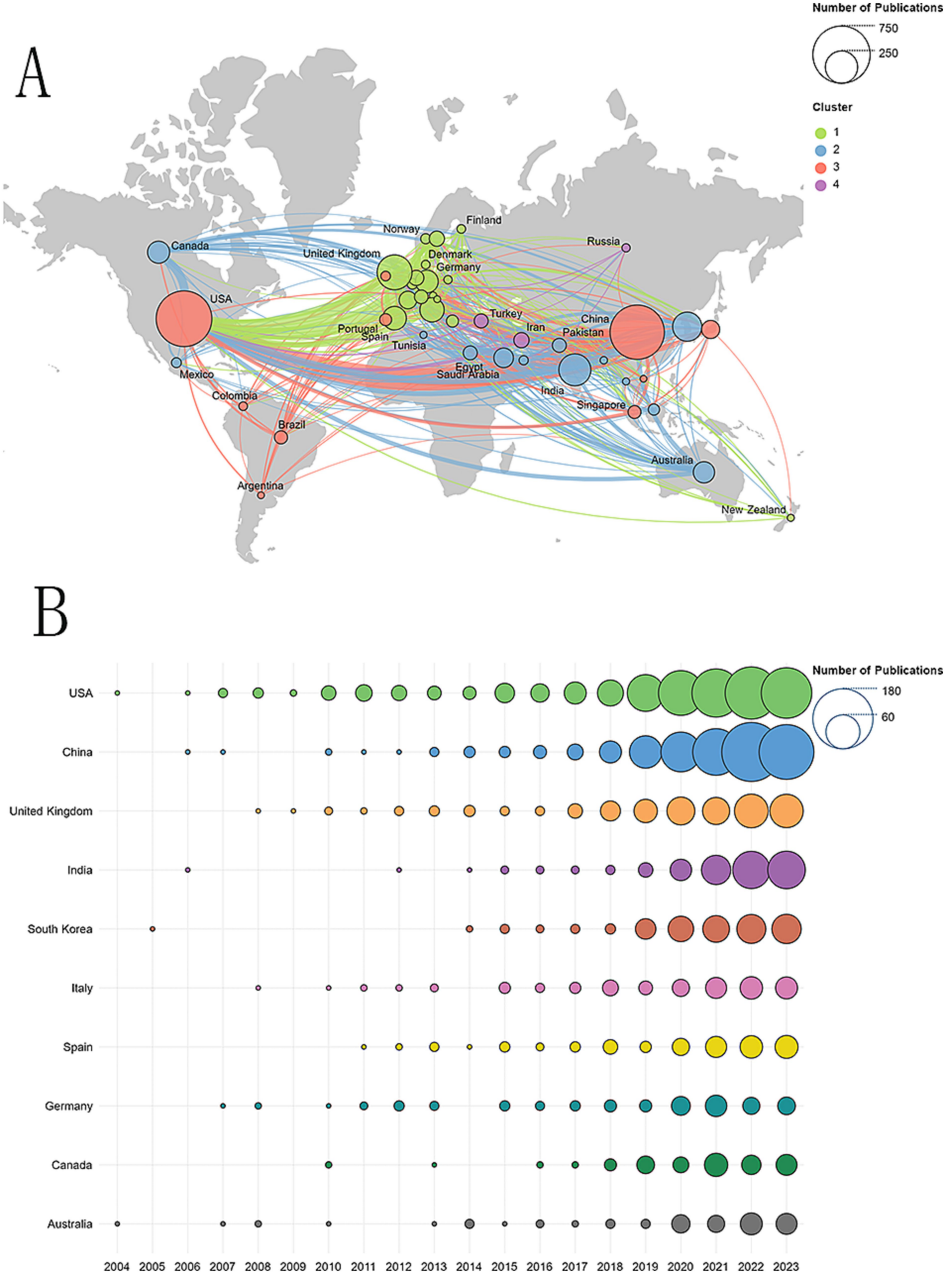
**(A)** Geographical distribution of collaborations among countries/regions. **(B)** Annual publication trends of the top 10 countries/regions.

A total of 84 countries/regions had published relevant articles in this field. Figure 3A shows an overview of the global distribution of these studies, with the circle size representing the number of publications in each country. The lines connecting the nodes indicate the collaboration between countries, and the thickness of the lines reflects the closeness of the cooperation, known as total link strength (TLS). As can be seen from the world map in Figure 3A, the countries with more than 200 published articles are, in order, the United States, China, the United Kingdom, and India. Figure 3B shows annual publication trends for the top 10 countries in research productivity over the last 20 years. Table 1 details the top 10 countries in research productivity in the previous 20 years. The United States topped the list with 672 articles, followed by China with 638 articles, then the United Kingdom (263 articles) and India (222 articles). From the perspective of cooperation, we can intuitively observe that the United States and China cooperated with most countries in this field. The United States has the most extensive partnerships, particularly with China, followed by the United Kingdom and Germany. The top five countries ranked by total link strength are the United States (587), the United Kingdom (474), China (336), Germany (244), and Italy (175). Regarding citations, the United States also holds the top position, while China lags significantly behind. Notably, although China ranks second in the total number of publications globally, its citation rate per paper is relatively low, which may be attributed to its shorter publication timeline. Since 2022, China has emerged as the leading country in terms of publications in this field, as shown in Figure 3B. This trend fully demonstrates China’s significant potential and growing importance in this field of research.

**Table 1:** Top 10 countries with most publication.

Rank	Country	Records	Citations	Average citations	TLS	Documentation density
1	United States	672	28,362	42.21	587	19.7
2	China	638	13,614	21.34	336	4.5
3	United Kingdom	263	9,549	36.31	474	38.4
4	India	222	3,637	16.38	157	1.5
5	South Korea	185	5,691	30.76	148	35.9
6	Italy	128	4,414	34.48	175	21.2
7	Spain	121	2,953	24.40	169	25.6
8	Germany	112	4,752	42.43	244	13.2
9	Canada	106	2,640	24.91	127	26.8
10	Australia	98	2,989	30.50	141	38.4

### 3.4 Contributions of top institutions

**Figure 4:**
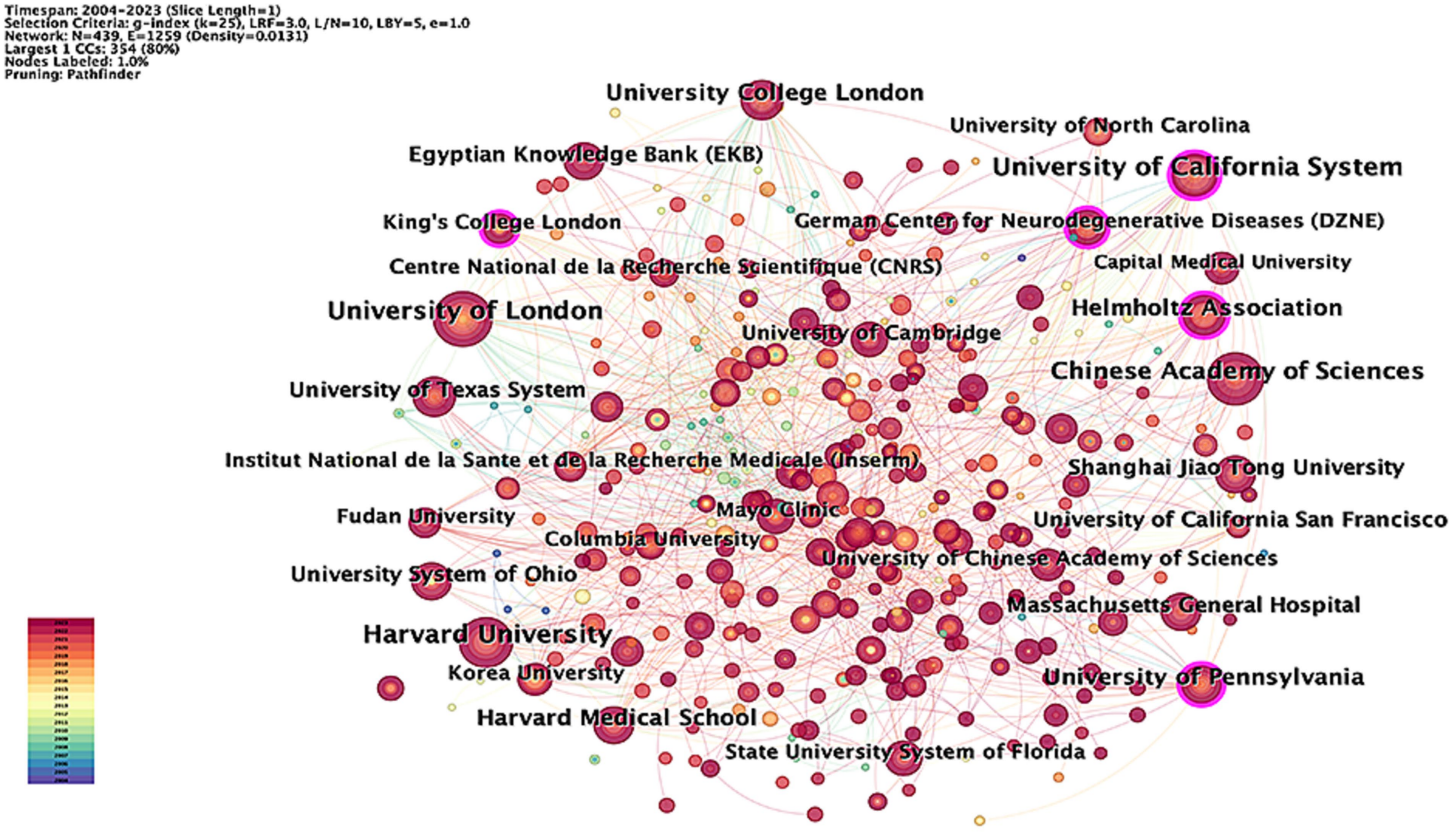
Network of institutions related to AI in AD.

Figure 4 presents a collaborative network graph of institutions engaged in research within this field, generated by the default settings of CiteSpace. In this graph, nodes represent the number of institutional publications, with larger nodes indicating more articles. The links between nodes illustrate collaborative relationships between institutions. Table 2 lists the top 10 productive institutions in the field. The University of London stands out as the most productive institution, with 80 publications, followed by the University of California System (74), Harvard University (74), and the Chinese Academy of Sciences (70). Regarding centrality, the higher-ranked institutions include the University of California System (0.11) and the University of Pennsylvania (0.11), followed by Harvard University (0.10), the Helmholtz Association (0.10), and University College London (0.06). The Chinese Academy of Sciences ranked seventh in centrality (0.04). Although it ranks third in terms of publication volume and exhibits high productivity, its lower centrality score indicates relatively limited international influence Consequently, the Chinese Academy of Sciences should focus on strengthening international cooperation and enhancing communication with other organizations. While the United States serves as the epicenter of research in this field, the University of London, located in the United Kingdom, emerges as a significant research institution, highlighting the UK’s considerable international influence in this area.

**Table 2:** Top 10 Institutions with most publication.

Rank	Institution	Records	Centrality
1	University of London	80	0.05
2	University of California System	74	0.11
3	Harvard University	74	0.1
4	Chinese Academy of Sciences	70	0.04
5	University College London	54	0.06
6	Harvard Medical School	47	0.04
7	University of Pennsylvania	45	0.11
8	Helmholtz Association	43	0.1
9	Massachusetts General Hospital	39	0.01
10	Egyptian Knowledge Bank (EKB)	39	0.03

### 3.5 Publication of authors

**Figure 5:**
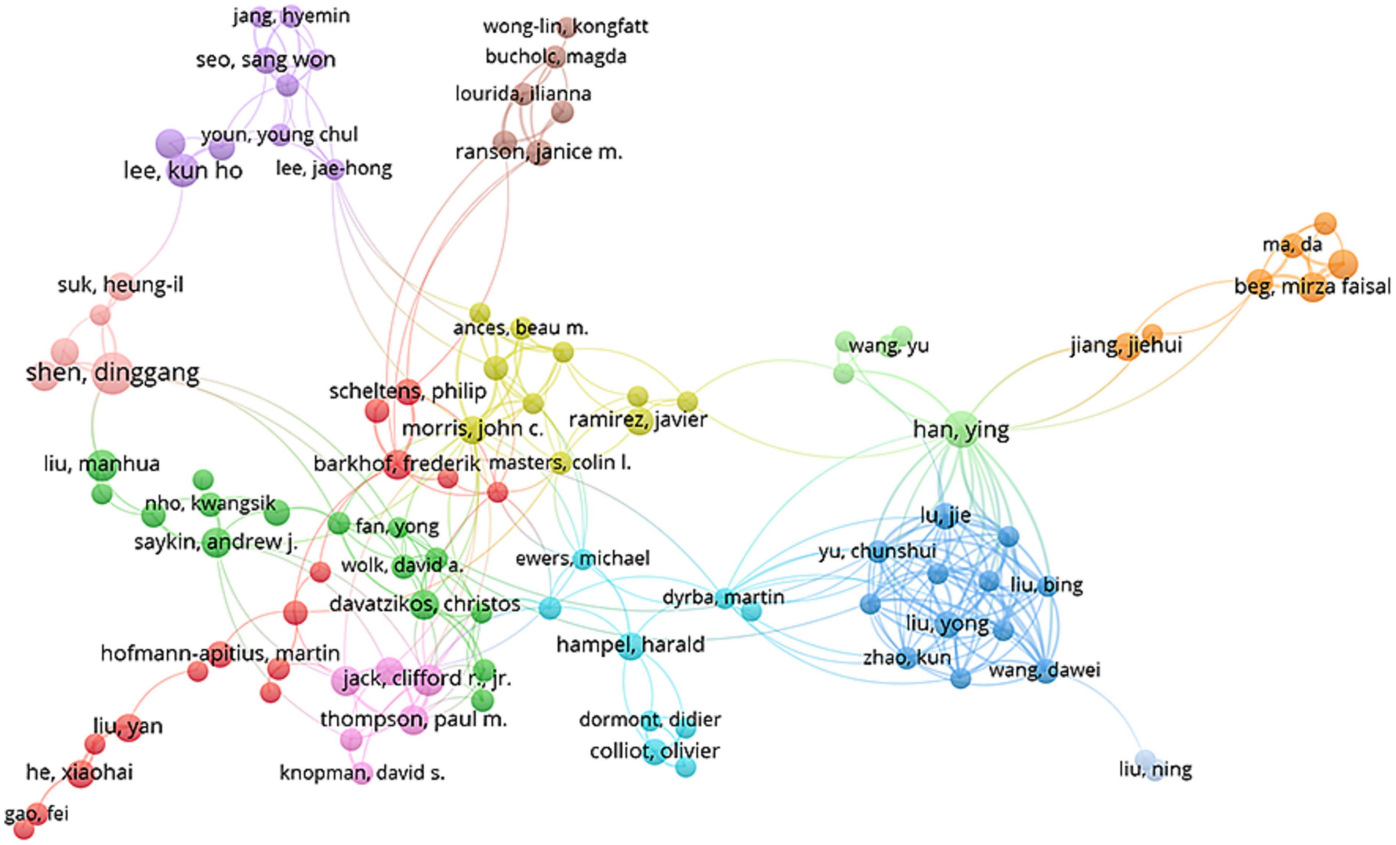
Network of authors related to AI in AD.

A total of 11,024 authors have contributed to research in this area. Among them, 103 authors have published more than six articles, as shown in Figure 5. Table 3 lists the top 10 authors biased on the number of publications in this field. The most prolific author is Shen Dinggang, who has published 23 articles. Following him are Han Ying and Adeli Hojjat, ranked second and third, respectively. In terms of average citations, Shen Dinggang remains in first place, followed by Jack Clifford R., Jr. and Adeli Hojjat. A combination of publication volume and average citations is crucial for assessing the productivity of authors in this field. Professor Shen, Dinggan was the founding dean of the School of Biomedical Engineering at the Shanghai University of Science and Technology and the director of the IDEA Lab (Image Display, Enhancement and Analysis Lab). He is recognized as one of the earliest researchers to explore the potential of artificial intelligence in medical imaging and has pioneered the application of deep learning to the analysis of brain development and diseases. Adeli, Hojaht was the second most prolific author, with research interests in biological and brain signal. Concerning collaboration, authors from different countries appear to be relatively weak. Therefore, authors in this field should strive to enhance international collaborations and foster broader communication with a larger network of researchers.

**Table 3:** Top 10 authors with most publication.

Rank	Author	Records	Citations	Average Citations	TLS
1	Shen, Dinggang	23	3,240	140.9	18
2	Han, Ying	16	405	25.3	29
3	Adeli, Hojjat	15	1,573	104.9	8
4	El-Sappagh, Shaker	13	380	29.2	10
5	Lee, Kun Ho	13	285	21.9	10
6	Jack, Clifford R., Jr.	12	1,305	108.8	30
7	Liu, Manhua	12	1,044	87.0	4
8	Barkhof, Frederik	11	454	41.3	13
9	Thompson, Paul M.	11	549	49.9	21
10	Abuhmed, Tamer	10	268	26.8	10

### 3.6 Contributions of top journals

**Figure 6:**
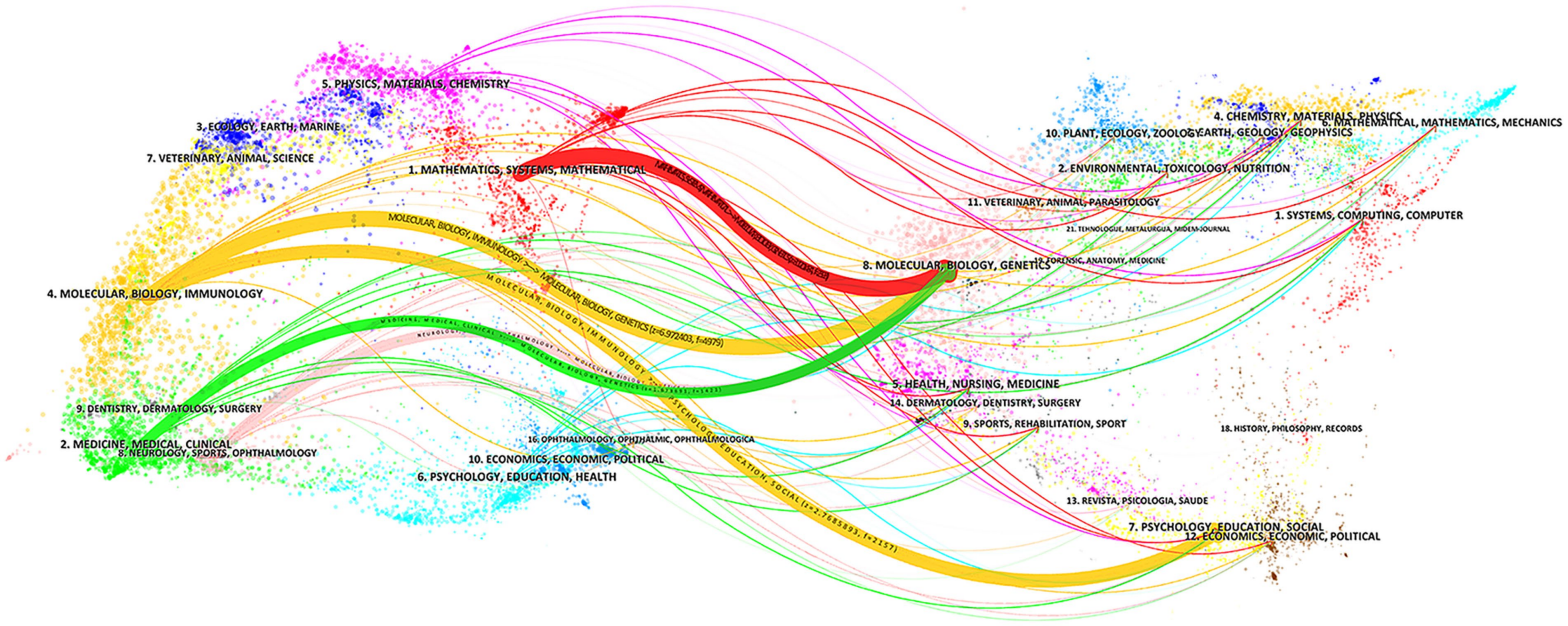
Double maps overlap of AI-based AD research journals conducted by CiteSpace.

A total of 531 journals have published the articles in question, with 48 of these journals releasing more than 10 issues. Table 4 presents the top 10 journals based on the number of articles published. The leading journals are the *Journal of Alzheimer’s Disease* (123 articles), *Frontiers in Aging Neuroscience* (118 articles), and *Scientific Reports* (63 articles). In terms of citations, the top three were Neuroimage, Journal of Alzheimer’s Disease, and Frontiers in Aging Neuroscience, in that order. Figure 6 shows the two stacked plots of the journals. The mapping is divided into two sections: the left side represents the citing journals, which represent the research frontiers, while the right side represents the cited journals, which reflect the research bases. The colored paths represent the citation relationships (Wan et al., 2023; Zhang et al., 2021). As shown in Figure 6, there are four distinct citation paths. The research themes of the citing journals can be broadly classified into four categories: (1) Mathematics, Systems, and Mathematical, (2) Molecular, Biology, and Immunology, (3) Medicine, Medical, and Surgery, and (4) Neurology, Sports and Ophthalmology. The cited journals’ research topics can be further divided into two main categories: (1) Molecular, Biology, and Genetics, and (2) Psychology, Education, and Social.

**Table 4:** Top 10 most productive journals.

Rank	Journals	Records	Citations	Average citations	IF (2022)	JCR (2022)
1	Journal of Alzheimers Disease	123	2,439	19.83	4.0	Q2
2	Frontiers in Aging Neuroscience	118	1,781	15.09	4.8	Q2
3	Scientific Reports	63	1,518	24.10	4.6	Q2
4	IEEE Access	56	957	17.09	3.9	Q2
5	Frontiers in Neuroscience	53	1,432	27.02	4.3	Q2
6	Neuroimage	46	5,422	117.87	5.7	Q1
7	Computers in Biology and Medicine	41	811	19.78	7.7	Q1
8	Alzheimers and Dementia	40	1,274	31.85	14	Q1
9	Plos One	40	1,303	32.58	3.7	Q2
10	Applied Sciences-Basel	34	208	6.12	2.7	Q2/Q3

### 3.7 Analysis of co-cited references

**Figure 7:**
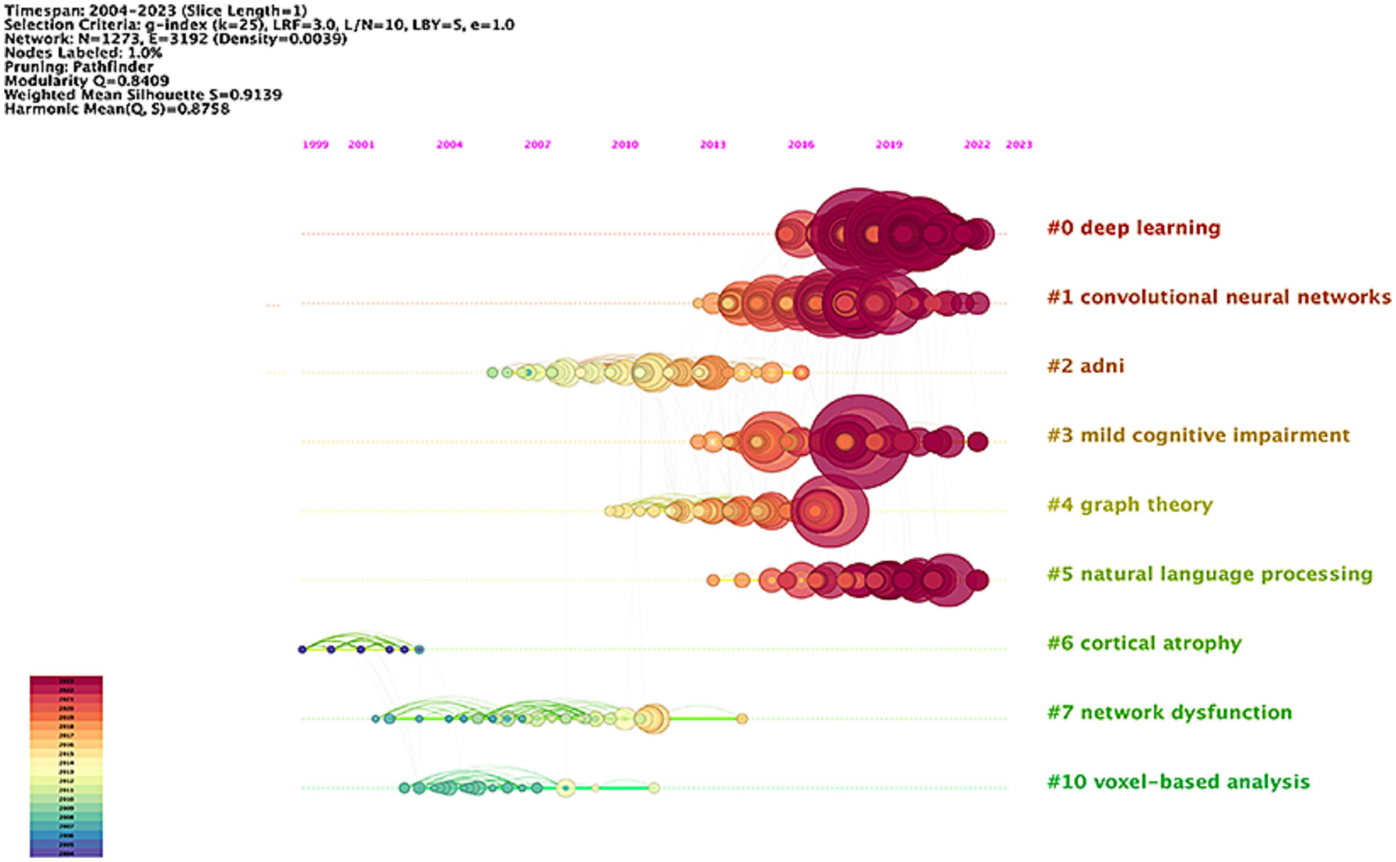
Network visualization map of the timeline view of co-cited literatures in the field of AI in AD.

Table 5 presents the 10 most highly cited literature in this research area, emphasizing early detection, diagnosis, disease course classification, and prediction of disease progression in AD. The most frequently cited paper, with 163 citations, was authored by Jack CR and published in *Alzheimer’s Dementia*. This was followed by an article from the Alzheimer’s Association, also in *Alzheimer’s Dementia*, which received 156 citations, and a study by Basaia S published in *Neuroimage-Clinical*, with 140 citations. Figure 7 shows the timeline view of the co-cited literature. The analysis reveals a Modularity Q of 0.8409 and a Silhouette S score of 0.9139, indicating a convincing clustering effect and excellent network homogeneity. The timeline view reflects trends in research hotspots over time. The clustering results categorize the research areas into 11 distinct clusters. Recent hotspots include deep learning (#0), convolutional neural networks (#1), mild cognitive impairment (#3), and natural language processing (#5). These areas are anticipated to remain focal points of research soon, signifying a growing interest among researchers in the application of artificial intelligence networks and precision medicine in AD.

**Table 5:** Top 10 articles according to number of citations.

Rank	Title	Journal	Author	Year	Citations
1	NIA-AA Research Framework: Toward a biological definition of Alzheimer’s disease	Alzheimers Dement	Jack CR	2018	163
2	2018 Alzheimer’s disease facts and figures	Alzheimers Dement	Alzheimers Assoc	2018	156
3	Automated classification of Alzheimer’s disease and mild cognitive impairment using a single MRI and deep neural networks	Neuroimage-Clin	Basaia S	2019	140
4	A review on neuroimaging-based classification studies and associated feature extraction methods for Alzheimer’s disease and its prodromal stages	Neuroimage	Rathore S	2017	102
5	Hierarchical Fully Convolutional Network for Joint Atrophy Localization and Alzheimer’s Disease Diagnosis using Structural MRI	IEEE T Pattern Anal	Lian CF	2020	100
6	Deep Learning in Alzheimer’s Disease: Diagnostic Classification and Prognostic Prediction Using Neuroimaging Data	Front Aging Neurosci	Jo T	2019	99
7	Convolutional neural networks for classification of Alzheimer’s disease: Overview and reproducible evaluation	Med Image Anal	Wen JH	2020	98
8	A parameter-efficient deep learning approach to predict conversion from mild cognitive impairment to Alzheimer	IEEE Neuroimage	Spasov S	2019	94
9	Multi-Modality Cascaded Convolutional Neural Networks for Alzheimer’s Disease Diagnosis	Neuroinformatics	Liu MH	2018	92
10	ImageNet Classification with Deep Convolutional Neural Networks	Communications of the ACM	Krizhevsky Alex	2017	90
10	A multi-model deep convolutional neural network for automatic hippocampus segmentation and classification in Alzheimer’s disease	Neuroimage	Liu MH	2020	90

### 3.8 Keyword co-occurrence and burst detection

**Figure 8:**
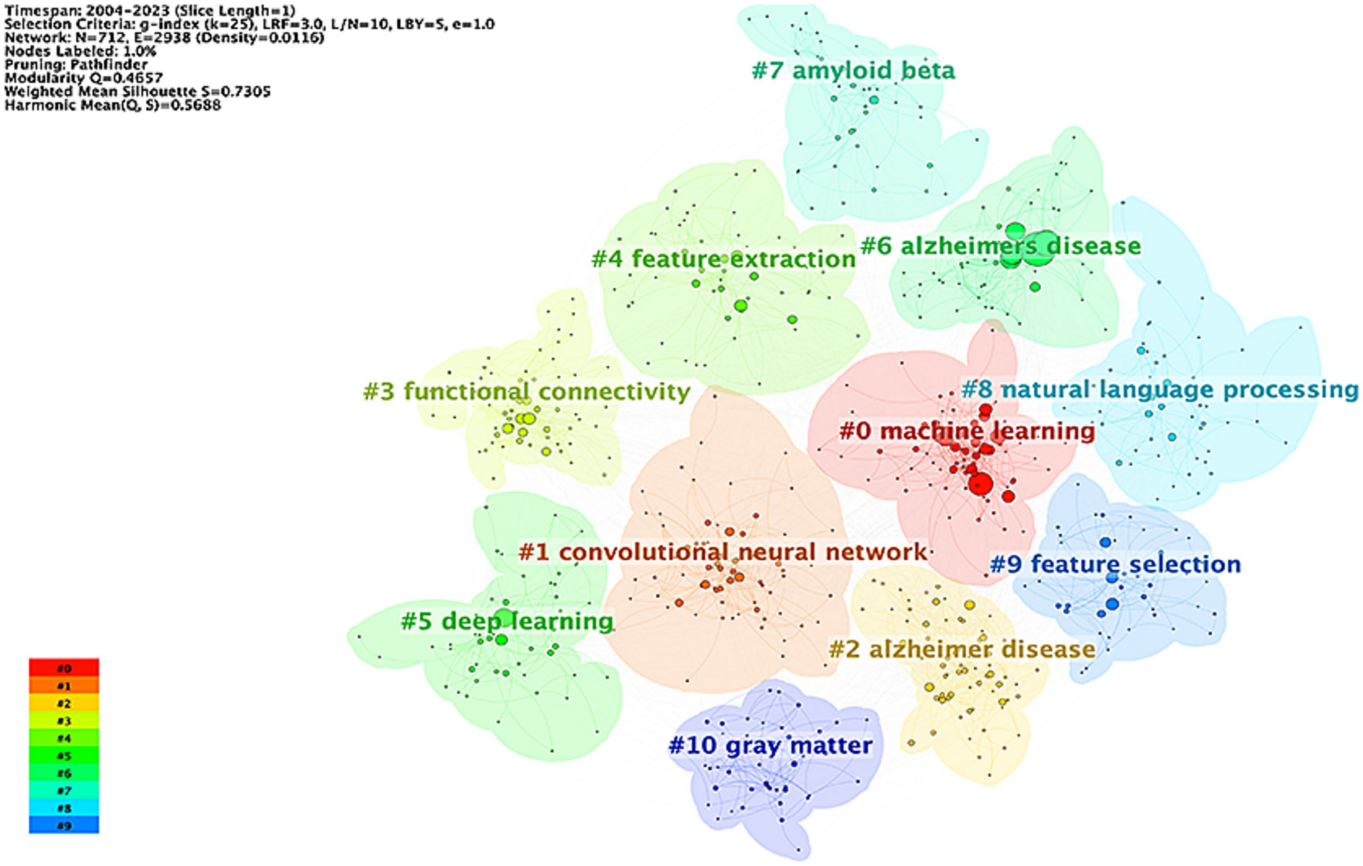
Keyword clustering knowledge graph in the field of AI in AD.

Keywords play a crucial role in emphasizing the core content of an article. Analyzing keywords allows for the identification of research hotspots and emerging trends within a particular field. In this study, we employed a clustering approach based on a knowledge graph, utilizing CiteSpace software to analyze keywords. The clustering results revealed that the research field could be divided into 11 clusters, as shown in Figure 8. The reasonableness and validity of these clustering results were determined based on modularity Q and Silhouette S. According to previous studies (Sabe et al., 2024), Q > 0.3 indicates a significant clustering structure, S > 0.5 indicates a reasonable clustering result, and S > 0.7 indicates a high degree of confidence in the clustering result. The results show that Q = 0.4657 > 0.3 and S = 0.7305 > 0.7, indicating that the keyword clustering results are both significant and reasonable. The top 5 clusters include: #0machine learning, #1 convolutional neural network, #2 Alzheimer disease, #3 functional connectivity, #4 feature extraction, #5 deep learning.

**Figure 9:**
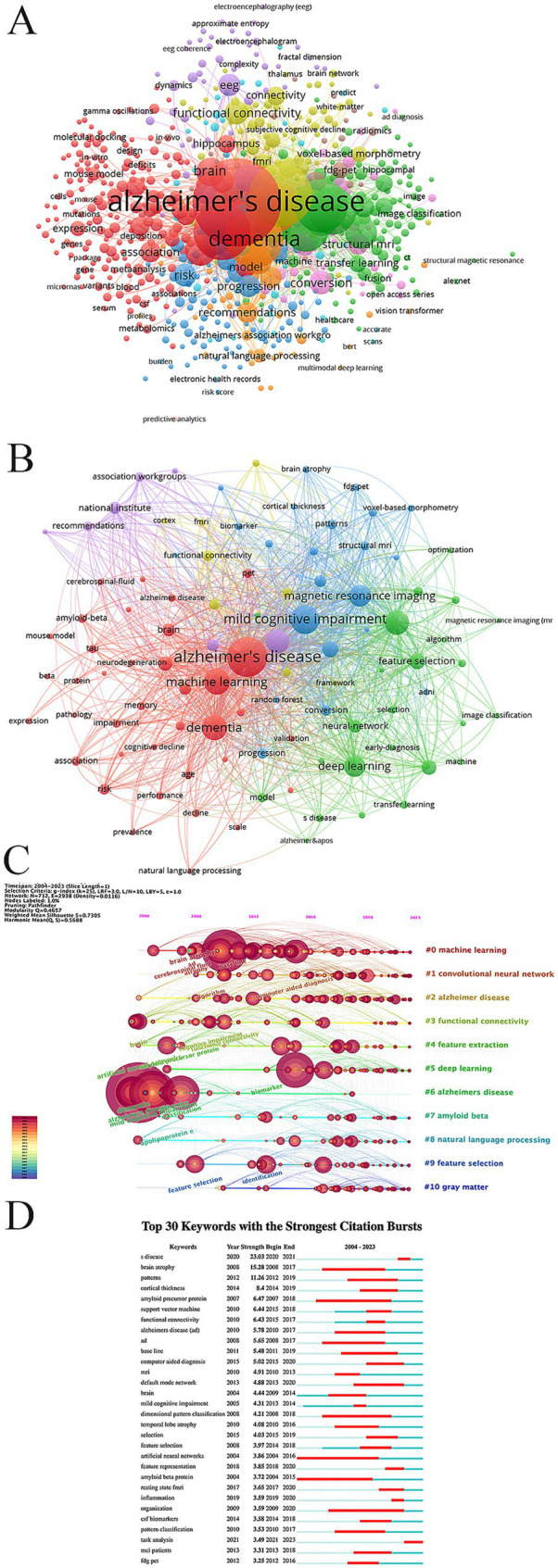
**(A)** Co-occurrence graph for the top 685 keywords with more than 5 occurrences. **(B)** Co-occurrence network map for the top 98 keywords. **(C)** Network visualization graph of the cluster timeline view of keywords in the field of AI in AD. **(D)** The top 30 keywords with the strongest citation burst.

The keyword co-occurrence map was obtained through using VOSviewer visual analysis software, analyzing a total of 2,316 articles. This analysis identified 7,125 keywords, of which 685 were high-frequency keywords, each appearing at least five times (Figure 9A). To provide a clearer understanding of the relationship between Alzheimer’s disease (AD) and artificial intelligence (AI), we further analyzed the initial 685 keywords, focusing on the 98 keywords with the highest frequency (Figure 9B). The results from Figure 9B allowed us to compile the top 50 hot keywords, as detailed in Table 6. A comprehensive analysis of occurrence frequency and TIS, excluding search terms from this study, we concluded that the most significant keywords linking AD and AI include classification, diagnosis, magnetic resonance imaging, prediction, feature selection, biomarkers, atrophy, functional connectivity, conversion, risk, progression, patterns, segmentation.

**Table 6:** Top 50 keywords in the field of AI for Alzheimer’s disease.

Rank	Keywords	Records	TLS	Rank	Keywords	Records	TLS
1	Alzheimer’s disease	1,582	7,340	26	Association workgroups	92	728
2	Mild cognitive impairment	810	4,683	27	Recommendations	91	722
3	Machine learning	664	3,545	28	Segmentation	91	583
4	Dementia	653	3,576	29	Structural MRI	90	596
5	Classification	585	3,243	30	Model	89	472
6	Diagnosis	524	3,109	31	Association	87	429
7	Magnetic resonance imaging	465	2,802	32	Pet	87	492
8	Deep learning	450	2,208	33	Cognitive impairment	86	461
9	Prediction	267	1,647	34	Support vector machine	80	509
10	Feature selection	243	1,448	35	Age	79	404
11	Convolutional neural network	214	1,116	36	Tau	79	417
12	Biomarkers	205	1,277	37	Memory	78	394
13	Brain	165	846	38	Neuroimaging	78	558
14	Neural-network	160	886	39	Amyloid-beta	74	341
15	National institute	148	1,108	40	Connectivity	72	418
16	Atrophy	145	952	41	Impairment	69	383
17	Artificial intelligence	140	785	42	Biomarker	65	418
18	Disease	140	725	43	Decline	65	383
19	Functional connectivity	115	619	44	Hippocampus	65	361
20	Conversion	114	885	45	Performance	65	377
21	Networks	109	525	46	Disease	63	349
22	Risk	108	574	47	Transfer learning	63	344
23	Progression	104	712	48	Computer-aided diagnosis	62	380
24	EEG	99	466	49	Images	62	375
25	Patterns	95	629	50	Identification	59	319

These represent the principal areas of interest concerning the application of AI in AD. Figures 9C,D provide an overview of the dynamic development and cutting-edge trends of AI in AD research, employing timeline analysis methods and keyword burst detection techniques.

## 4 Discussion

### 4.1 General information

In this study, we conducted an exhaustive review of the literature on AI in AD published between 2004 and 2023, utilizing the WOSCC database. After a rigorous screening process with inclusion and exclusion criteria, we identified a total of 2,316 high-quality papers. These papers were co-authored by 11,024 researchers from 439 institutions in 84 countries/regions and were published in 531 academic journals, providing a robust data foundation for our study.

The volume of scientific literature produced is often regarded as a principal indicator for assessing the developmental dynamics of a specific subject area. This study meticulously analyzes the research dynamics of the application of AI in AD from 2004 to 2023. Our findings indicate a consistent growth trend in the volume of literature in this field, with a notable 88% of the total publications occurring in the last 6 years. This remarkable growth can largely be attributed to the rapid advancements of DL. Further analysis reveals a sharp rise in publication volume since 2018, primarily driven by the proposal and practical application of various novel deep learning frameworks (Liu S. Q. et al., 2015; Qiu et al., 2020). Among these innovations is a framework capable of generating high-resolution disease probability maps with diagnostic accuracy comparable to that of neuroscientists, achieved by fusing a fully convolutional network (FCN) with a multilayer perceptron (MLP). Currently, the application of AI in AD is a topic of considerable interest in clinical medical research, and it is expected to have significant research potential and diverse applications in the future.

A review of the global distribution of research on AI in the field of AD revealed that the United States holds a prominent position, with a total of 672 relevant papers published, accounting for 29.0% of the global total. This makes it the country with the highest number of publications in this field. China follows closely, having published 638 papers, which constitutes 27.5% of the total. Together, these two countries account for 56.6% of all papers published, underscoring their leadership at the intersection of AI and AD research. However, when comparing research outputs across countries, we must consider differences in the size of their populations. To standardize the number of publications across countries, we considered counting the percentage of scientific practitioners in each country, as this might make the results more accurate. However, due to the greater difficulty of counting the number of practitioners in specific disciplines, we ultimately decided to use the total number of people in a country as the basis for our calculations. For this purpose, we defined a new metric, publication density, calculated as the total number of publications in a country divided by the country’s total population (population in tens of millions), as shown in Table 1. Despite the notable research achievements of the United States and China in this area, other countries such as the United Kingdom and Australia stand out in the ranking of the density of issuance after taking into account the population factor, and they are in fact among the countries with the highest efficiency of article output (i.e., the number of articles per 10 million population), demonstrating a very high capacity to produce articles. Moreover, despite China’s significant research output in this field, its average citation rate of papers is concerning, ranking second to last among the top 10 countries in terms of productivity. This phenomenon may be related to the relatively recent initiation of research in this field in China and the shorter timeframe for publication. Nevertheless, it is noteworthy that China is the fastest-growing country in terms of published papers and has now risen to become the second-largest contributor globally, fostering a solid bridge of cooperation with the United States, a leader in the field. As technology advances and research deepens, it is anticipated that an increasing number of countries and researchers will engage in interdisciplinary research on AD and AI. This will facilitate the advancement of academic knowledge and the practical application of this field.

In the global scientific research cooperation network, the United States serves as a central hub, maintaining close partnerships with scientific and technological powers such as China, United Kingdom, Germany and Canada. However, the current cooperation pattern exhibits a clear geographical concentration, primarily within North America, Europe, and some Asian countries. To drive further progress in scientific research, it is essential to expand the scope of international cooperation, especially by strengthening research ties with developing nations. At the same time, financial support and research funding remain crucial catalysts for scientific advancement, playing a key role in fostering scientific productivity. By increasing investment and incentives for research, a broader range of countries can be empowered to participate, thus enhancing the global impact of scientific endeavors and positioning research networks as vital contributors to scientific progress worldwide.

At the institutional level, the United States holds five of the top 10 positions in terms of research productivity. Leading the list is the University of London, with a high output of 80 articles, followed by the University of California System and Harvard University, each contributing 74 articles. The Chinese Academy of Sciences also demonstrates substantial research capacity, with 70 published papers. These figures clearly reflect the United States’ significant advantage in overall publication volume, while British institutions likewise exhibit notable scientific strength. However, despite the University of London’s leading publication count, its centrality index is relatively low at 0.05. In contrast, Harvard University and the University of California System have centrality indices of 0.11 and 0.1, respectively, indicating that they occupy a more central position in the academic exchange network and have a wider range of external cooperation and exchange. Similarly, although the Chinese Academy of Sciences has a high number of publications, its centrality index of 0.04 indicates a relative lack of international cooperation and academic exchanges. These findings highlight the importance of enhancing global academic collaboration. In summary, research activities in the United States, China, and the United Kingdom stand out on the global scale. U.S. institutions, particularly the University of California System and Harvard University, lead in both research output and influence. Meanwhile, the UK and China also demonstrate significant research strength. To elevate their academic status, institutions across nations must strengthen their international collaborations to advance scientific research further.

The analysis of core authors shows that most of the top 10 scholars hail from the United States and China, highlighting the outstanding contributions of these two countries in specific research areas. This finding aligns with previous institutional analyses, reinforcing the academic leadership of both nations. Among them, Shen Dinggang leads in number of publications, followed by Han Ying and Adeli Hojja. Notably, although Jack, Clifford R., Jr. has published fewer articles, he boasts the second-highest average citation count, trailing only Shen, Dinggang. This demonstrates their profound academic influence. Figure 5 further illustrates the central positions of Shen, Dinggang, and Jack, Clifford R., Jr. within the academic network, suggesting that their roles as key links between research clusters may be a crucial factor in their high citation index. However, Figure 5 also reveals the limitations of current research collaborations, as many research teams remain confined to domestic partnerships. This highlights the urgent need to enhance international collaboration, which is critical to advancing research progress and innovation in the diagnosis and treatment of disease.

The assessment of journal quality typically relies on several core metrics, including the impact factor (IF), JCR journal classification, and overall citation frequency. A review of the core journals in the field reveals that Neuroimage ranks highest in terms of both total citations and impact factor (IF), with a JCQ partition of Q1, indicating its position as a leading journal in the study of AI applications AD research. Focusing on these high-impact core journals offers crucial insights into the latest developments in the field. The range of impact factors, spanning from 2.7 to 14, demonstrates that research results in AI applied to AD have the potential to be published in top journals. Meanwhile, the *Journal of Alzheimer’s Disease* has published the most relevant articles, underscoring its central role in the field.

Co-citation analysis is an effective tool for measuring relationships between articles or authors, serving as a key indicator of scholarly impact (Wang et al., 2017). Citation and co-citation analysis are foundational elements of bibliometric research, enabling identifying influential literature and assessing scientific progress. Frequently cited articles are often emblematic of high-quality, innovative research with a significant impact on a particular field. Table 5 summarizes the 10 most frequently cited papers in this domain. It is important to note that most of the literature with higher citation counts is newer literature, and older literature may be at an inherent disadvantage. This may be since as the academic field evolves and the focus of research changes, new research results emerge, making it easier for newer literature to gain attention and citations, often putting older literature at a disadvantage in the citation competition. Although older literature may not stand out in terms of citations, it plays an equally important role in terms of its far-reaching impact on the field of study and innovation in research methodology. The most cited study, authored by Jack et al. (2018), proposed the NIA-AA research framework for the biological definition of AD, which outlines the disease’s various stages using biomarkers. This framework is critical for the accurate diagnosis and early intervention of AD and has become a cornerstone for further research. Second most frequently cited article, published by Alzheimer’s Association (2018), emphasizes the importance of early diagnosis. The third most cited article, authored by Basaia et al. (2019), validated a deep learning algorithm that predicts AD and mild cognitive impairment (c-MCI) based on structural MRI scans, offering a powerful tool for automated diagnosis. Other highly cited papers (Jo et al., 2019; Lian et al., 2020; Liu M. H. et al., 2018; Liu et al., 2020; Rathore et al., 2017; Spasov et al., 2019; Wen et al., 2020) focus primarily on the application of deep learning, particularly convolutional CNN, in AD diagnosis. These studies highlight the significant potential of AI and deep learning in improving diagnostic precision through the integration of multi-modal neuroimaging data. For example, Liu M. H. et al. (2018) proposed a cascaded CNN model that integrates MRI and PET data to provide a comprehensive analysis of structural and functional brain alterations in AD patients. Similarly, Liu et al. (2020) developed an automatic hippocampus segmentation method using multi-model deep CNNs, significantly enhancing diagnostic accuracy. The article discusses in depth the prediction of conversion from MCI to AD (Spasov et al., 2019), has important implications for early intervention and treatment. In conclusion, current AD research mainly concerns the application of DL in AD diagnosis, the fusion of multi-modal neuroimage data, the prediction of the transformation from MCI to AD, and automatic hippocampus segmentation and classification. These studies offer novel methodologies and insights into the early diagnosis and personalized treatment of AD, making significant contributions to advancing clinical diagnosis and disease management.

### 4.2 Research hotspots and emerging trends

#### 4.2.1 Research hotspots

A keyword co-occurrence analysis provides an accurate representation of the core themes, emerging hotspots, and development trends within a specific academic field (Wu et al., 2021). This analysis offers valuable insight into tracking scientific progress and identifying research hotspots. After a comprehensive analysis of the frequency of occurrence and TIS (Table 6), the most important keywords linking AD and AI can be categorized into three themes: (1) Disease classification and progression: Alzheimer’s disease, mild cognitive impairment, and dementia. (2) AI and ML technology: machine learning, deep learning, convolutional neural network, neural network, artificial intelligence. (3) The function of ML: classification, diagnosis, prediction (including disease conversion, risk, and disease progression), feature selection, functional connectivity, patterns, and segmentation.

Moreover, the timeline visualization based on the co-occurrence keyword network (Figure 9C) illustrates the trajectory of AI in AD research. Initially, the focus was primarily on clinical applications (Kloeppel et al., 2008; Kohannim et al., 2010; Liu L. et al., 2015; Swarup and Geschwind, 2013; Zeng and Wu, 2016). However, with the continuous advancement of AI technology and the innovation in treatment methods, research emphasis has gradually shifted toward emerging areas such as early detection (Hu et al., 2023; Ma et al., 2023; Mahendran and Vincent, 2022), risk assessment (Akter et al., 2022; Jiang et al., 2022; Park et al., 2020), and prognosis prediction (Jo et al., 2019; Liu X. et al., 2018; Velazquez and Lee, 2022). From the above keyword analysis, two main research directions in the field of AD and AI emerge (1) Accurate identification of AD phases and early diagnosis of AD; (2) Risk assessment and prediction of disease progression in AD.

Accurate identification of AD phases and early diagnosis of AD: The progression of AD is complex, encompassing multiple stages. Individuals diagnosed with mild cognitive impairment (MCI) face an elevated risk of developing AD, with a conversion rate of approximately 15% per year (Davatzikos et al., 2011). Consequently, early, and accurate diagnosis is crucial for slowing disease progression and improving treatment outcomes (Grundman et al., 2004). Initially, researchers focused on AD classification and diagnosis using traditional ML techniques, often with healthy individuals as controls (Chen et al., 2011; Khazaee et al., 2015; Magnin et al., 2009). Some proposed a two-stage classification, distinguishing MCI from AD (Khazaee et al., 2017; Wang et al., 2018). However, traditional ML techniques often rely on manual feature extraction, a time-consuming process that complicates data analysis (Plis et al., 2014). In recent years, DL models have shown significant potential in handling high-dimensional neuroimaging data with their powerful feature extraction and classification capabilities (Mohsen et al., 2018; Wagner et al., 2021). Researchers started applying DL methodologies to achieve precise AD diagnosis and early prediction. (Guo and Zhang, 2020) developed an improved DL algorithm (IDLA) that integrated clinical text data (including age, sex, genes, etc.) for the objective AD classification. Odusami et al. (2021) proposed a fine-tuned ResNet18 network for classification tasks, achieving high accuracy in distinguishing early MCI from AD, late MCI from AD, and MCI from early MCI, with accuracy rates of 99.99, 99.95, and 99.95%, respectively (Odusami et al., 2021). Loddo et al. (2022) introduced a novel deep integration method that fused the predictive advantages from multiple models, improving classification accuracy and robustness. This innovation provided new approaches for early AD diagnosis. Notably, Alorf and Khan (2022) proposed a novel method for multi-label classification of the six stages of AD using the brain’s functional connectivity network, achieving high accuracy. This study not only expanded the scope of AD classification but also provided a new perspective for identifying biomarkers related to the disease. In summary, the application of AI in AD research is becoming increasingly advanced, offering substantial support for early diagnosis, precise treatment, and prognosis prediction of the disease.

Risk assessment and prediction of disease progression in AD: As shown in Figure 6, the keywords “prediction,” “conversion,” “risk,” and “progression” demonstrate the potential of AI in AD risk assessment and disease progression prediction. A substantial body of research has indicated that AD-related pathophysiological changes occur well before clinical symptoms appear, emphasizing the need for early interventions (Sperling et al., 2014). In the absence of efficacious pharmacological treatments, are crucial to reducing the risk, delaying onset, or slowing the pathological progression of AD (Vermunt et al., 2019). AI-based risk prediction models offer a new strategy for AD management and prevention. For example, You et al. (2022) developed a novel AD risk prediction model, UKB-DRP, which applied ML techniques to a set of 366 features (genetic and environmental factors), identifying 10 key predictors such as age and ApoE ε4. The model successfully predicted AD risk over 5 years, 10 years, or even longer. Forecasting disease progression from MCI to AD has also emerged as a crucial research area (Grant et al., 2018; Ward et al., 2013). In 2019, Lee et al. developed a comprehensive framework that integrated longitudinal multidomain data, achieving up to 81% accuracy in predicting MCI conversion to AD (Lee et al., 2019). More recently, Wang et al. (2024) developed an innovative DL model integrating multi-modal data and interaction effects. This model was able to accurately predict the conversion from MCI to AD within 4 years with an accuracy of 92.92%.

In addition, a growing number of studies have begun to focus on the role of environmental factors in the development of AD. For example, Fania et al. (2024) used machine learning and interpretable artificial intelligence (XAI) methods to investigate the association between AD mortality and socioeconomic and health indicators of pollution, and found a strong association between pollutants such as ozone (O3) and nitrogen dioxide (NO2) and AD. These pollutants may affect the pathogenesis of AD through various pathways, such as triggering neuroinflammation, oxidative stress, etc., thereby accelerating the pathological process of AD (Becker et al., 2002). This link between the environment and human health is particularly important in the context of One Health. Artificial intelligence technologies can integrate environmental data (e.g., air quality monitoring data) with other data, such as clinical and genetic data, to assess an individual’s risk of developing Alzheimer’s disease more fully and to inform the development of targeted prevention strategies. For example, by monitoring the levels of environmental pollutants in a particular area and combining this with information on the genetic background and lifestyle of the residents, AI models can be used to predict the potential risk of developing Alzheimer’s and interventions such as improving air quality and adjusting the living environment can be taken in advance, which is expected to reduce the incidence rate of Alzheimer’s or slow the process of its onset. This further demonstrates the promise of AI in AD research, which is not limited to the diagnosis and treatment of the disease, but also extends to the prevention of the disease and the comprehensive consideration of environmental factors.

#### 4.2.2 Emerging trends

The keyword burst detection algorithm is designed to track the significant popularity in keywords popularity over a specific period, providing an effective tool for identifying hot topics. Burst keywords are essential indicators of cutting-edge research hotspots, offering insights into emerging trends. Notably, “task analysis” is projected to become a key hotspot starting from 2021 (Figure 9D). “Task analysis” involves breaking down complex tasks into smaller, more manageable subtasks for systematic and refined analysis. This trend reflects a growing interest in enhancing model performance for more accurate early diagnosis and prediction of diseases (Gao et al., 2022; Jeon et al., 2023; Mitra and Ul Rehman, 2024; Nan et al., 2024; Song et al., 2021). Simultaneously, it demonstrates the increasing focus on developing intelligent assessment tools capable of accurately predicting disease progression (Nguyen et al., 2024) and thoroughly evaluating a patient’s responses to various tasks (El-Gamal et al., 2020; Kim et al., 2022; Palacios-Navarro et al., 2022). These advancements aim to provide robust data support for creating personalized treatment strategies. In conclusion, “task analysis” represents a significant keyword in AI research related to AD, exemplifying the ongoing evolution and advancement of research methodologies in this field. With the continued advancement of technology and deeper research, further breakthroughs in early disease detection, accurate diagnosis, effective treatment, predictive accuracy, and better care and prognosis are anticipated.

### 4.3 Limitations

There are some limitations in this study. First, due to the inherent limitations of bibliometric tools and databases, it was not feasible to integrate and analyze data from multiple databases or languages effectively. Consequently, the analysis is limited to the WoSCC database, and the justification for selecting this database is provided in the methodology section. Secondly, this study focuses solely on English-language literature, excluding academic results in other languages. Furthermore, the literature review spans 2004 to 2023, which may have restricted the consideration of earlier or more recent studies. Consequently, there may be some omissions or biases in the data. To enhance the comprehensiveness and accuracy of future studies, it is recommended to expand data sources to include multilingual literature and a wider range of literature types.

## 5 Conclusion

The findings of this study indicate a marked increase in the number of publications and citations in this field, reflecting the growing interest and attention from the academic community. The United States and China are leading the research efforts, highlighting the importance of continued international collaboration and exchange, especially among Asian countries and institutions. Strengthening these partnerships will be crucial for further advancements in this field. Our analysis also identifies key research areas, including the identification of different stages of AD, early diagnostic screening, risk prediction, and forecasting disease progression. Future research will explore AI methods, particularly task analysis, emphasizing integrating multimodal data and utilizing deep neural networks. These approaches aim to identify emerging risk factors, such as environmental influences on AD onset, predict disease progression with high accuracy, and support the development of prevention strategies. Ultimately, AI-driven innovations will transform AD management from a progressive, incurable state to a more manageable and potentially reversible condition, thereby improving healthcare, rehabilitation, and long-term care solutions.
